# Y1 and Y5 Receptors Are Both Required for the Regulation of Food Intake and Energy Homeostasis in Mice

**DOI:** 10.1371/journal.pone.0040191

**Published:** 2012-06-29

**Authors:** Amy D. Nguyen, Natalie F. Mitchell, Shu Lin, Laurence Macia, Ernie Yulyaningsih, Paul A. Baldock, Ronaldo F. Enriquez, Lei Zhang, Yan-Chuan Shi, Serge Zolotukhin, Herbert Herzog, Amanda Sainsbury

**Affiliations:** 1 Neuroscience Research Program, Garvan Institute of Medical Research, Darlinghurst, Sydney, New South Wales, Australia; 2 Bone and Mineral Research Program, Garvan Institute of Medical Research, Darlinghurst, Sydney, New South Wales, Australia; 3 Division of Cell and Molecular Therapy, University of Florida, Gainesville, Florida, United States of America; 4 Faculty of Medicine, University of New South Wales, Kensington, Sydney, New South Wales, Australia; 5 School of Medical Sciences, University of New South Wales, Kensington, Sydney, New South Wales, Australia; 6 Sydney Medical School, The University of Sydney, Sydney, New South Wales, Australia; University of Cordoba, Spain

## Abstract

Neuropeptide Y (NPY) acting in the hypothalamus is one of the most powerful orexigenic agents known. Of the five known Y receptors, hypothalamic Y1 and Y5 have been most strongly implicated in mediating hyperphagic effects. However, knockout of individual Y1 or Y5 receptors induces late-onset obesity – and Y5 receptor knockout also induces hyperphagia, possibly due to redundancy in functions of these genes. Here we show that food intake in mice requires the combined actions of both Y1 and Y5 receptors. Germline Y1Y5 ablation in Y1Y5^−/−^ mice results in hypophagia, an effect that is at least partially mediated by the hypothalamus, since mice with adult-onset Y1Y5 receptor dual ablation targeted to the paraventricular nucleus (PVN) of the hypothalamus (Y1Y5^Hyp/Hyp^) also exhibit reduced spontaneous or fasting-induced food intake when fed a high fat diet. Interestingly, despite hypophagia, mice with germline or hypothalamus-specific Y1Y5 deficiency exhibited increased body weight and/or increased adiposity, possibly due to compensatory responses to gene deletion, such as the decreased energy expenditure observed in male Y1Y5^−/−^ animals relative to wildtype values. While Y1 and Y5 receptors expressed in other hypothalamic areas besides the PVN – such as the dorsomedial nucleus and the ventromedial hypothalamus – cannot be excluded from having a role in the regulation of food intake, these studies demonstrate the pivotal, combined role of both Y1 and Y5 receptors in the mediation of food intake.

## Introduction

A major obstacle in the treatment of overweight and obesity is hunger. Loss of as little as 6–14% of body weight by energy restriction, with or without exercise, in obese men and women significantly increases appetite [1]. This increase in appetite is a significant predictor of subsequent weight regain in humans [2] and in diet-induced obese rats [3]. Moreover, people carrying obesity-risk genetic polymorphisms consistently show increases in appetite or measured food intake [4]. In light of the key role of appetite in determining energy balance, interventions that reduce hunger could enable more people to attain and maintain a healthy body weight.

Neuropeptide Y (NPY), a 36-amino acid peptide abundantly expressed in the central and peripheral nervous systems, is one of the most powerful orexigenic agents known. Continuous administration of NPY to the hypothalamus of normal animals results in massive hyperphagia and obesity [5], [6]. Importantly, many other orexigenic and anorexigenic agents – such as leptin, glucocorticoids, melanocortins and ghrelin – mediate effects on food intake and energy balance at least partially via the NPY-ergic system [7]–[9]. This makes NPY and its receptors (Y1, Y2, Y4, Y5 and – in mice – y6) possible targets for anti-obesity therapies.

Y1 and Y5 receptors have been strongly implicated in mediating the hyperphagic effects of NPY or energy restriction [10]–[12]. However, whereas male and female germline Y1 receptor deficient mice exhibit reduced fasting-induced food intake, they exhibit slight or no reductions in total daily food intake or NPY-stimulated feeding, and they also develop late-onset obesity [13]–[16]. Paradoxically, male and female germline Y5 receptor knockout mice are obese with increases in total daily food intake, fasting-induced food intake, body weight and adiposity, and they are not protected against leptin-deficiency-induced obesity [17], [18]. Additionally, chronic intracerebroventricular NPY administration to Y1 or Y5 receptor knockout mice induces a similar hyperphagic obesity syndrome as that seen in wildtype mice [6]. These observations might be attributed to compensatory effects of germline gene deletion. Indeed, germline Y5 receptor knockouts display exacerbated fasting-induced increases in hypothalamic expression of the orexigenic NPY and agouti related peptide (AgRP), and exacerbated decreases in that of proopiomelanocortin (POMC, which produces the anorexigenic α-melanocyte stimulating hormone, α-MSH) and the anorexigenic cocaine and amphetamine-related transcript (CART) [18]. However, when conditional deletion of hypothalamic Y1 receptors was induced in adult mice in order to circumvent possible compensatory effects, marked and significant effects on nesting and food scattering behaviours were observed, but there was no effect on total daily food intake or fasting-induced food intake [15]. Taken together, these findings suggest redundancies between Y1 and Y5 receptors in the control of energy homeostasis, in keeping with the observation that the genes are coordinately regulated by the same promoter region [19] and are co-expressed in the same neurons [20]–[22].

In order to circumvent the possible redundancy between Y1 and Y5 receptors in the control of food intake and energy homeostasis, we generated Y1Y5 receptor double knockout mice. Moreover, in order to investigate the possible role of hypothalamic Y1 and Y5 receptor signaling in the regulation of these processes, we also generated conditional Y1Y5 receptor double knockout mice (Y1Y5^lox/lox^) in which Y1 and Y5 receptors can be deleted in one step in adult mice by injection of an adeno-associated viral vector (rAAV) expressing Cre-recombinase. These germline and adult-onset hypothalamus-specific Y1Y5 receptor knockout mice were studied on a chow diet and on a high fat diet in order to determine the role of Y1 and Y5 receptors in the regulation of energy homeostasis.

**Figure 1:**
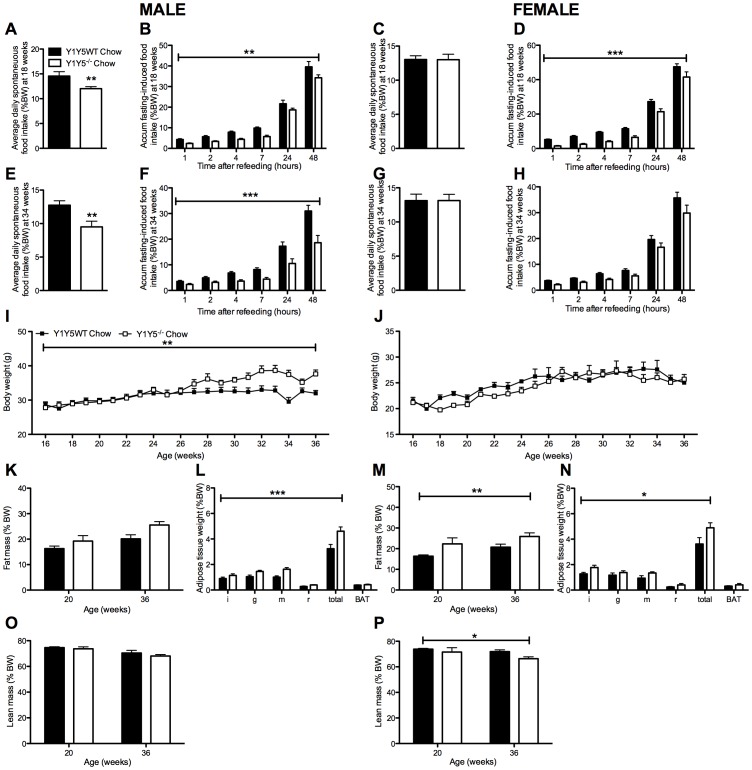
Increased body weight/adiposity despite reduced food intake in Y1Y5^−/−^ mice on chow. (A–H) Spontaneous (A, C, E, G) and accumulated (accum) 24-hour fasting-induced food intake (B, D, F, H), normalised to body weight, in male and female germline Y1Y5 receptor knockout (Y1Y5^−/−^) and wildtype control (WT) mice at 18 and 34 weeks of age. (I–J) Body weight from 16 to 36 weeks of age in male (I) and female (J) Y1Y5^−/−^ or WT mice. (K–P) Fat and lean masses as a percentage of body weight (%BW), measured using dual energy x-ray absorptiometry, in male (K, O) and female (M, P) Y1Y5^−/−^ and WT mice at 20 and 36 weeks of age. Weight (as %BW) of dissected white (WAT) and brown adipose tissue (BAT) depots at the end of the study in male (L) and female (N) Y1Y5^−/−^ and WT mice at 36 weeks of age. Abbreviations: i, inguinal; g, gonadal; m, mesenteric; r, retroperitoneal; total, summed weight of i, g, m and r WAT depots. Plotted values are means ± SEM of 5–12 standard chow-fed mice per group. *P<0.05, **P<0.01 or ***P<0.001 for Y1Y5^−/−^ versus WT mice of the same age and sex.

**Figure 2:**
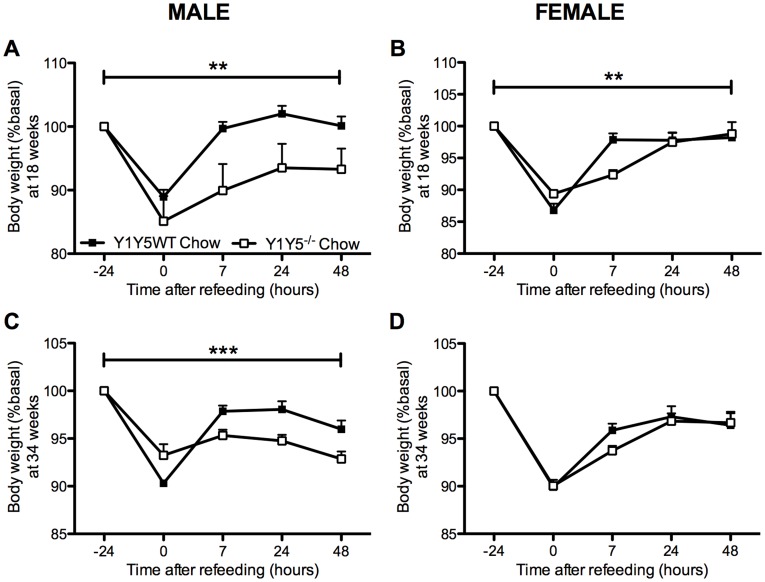
Slower weight regain after a 24-hour fast in Y1Y5^−/−^ mice on chow. Body weight measurements were taken at the time points of fasting-induced food intake measurements in male (A, C) and female (B, D) germline Y1Y5 receptor knockout (Y1Y5^−/−^) or wildtype control (WT) mice, at both 18 and 34 weeks of age. Data are presented as a percent of pre-fasting body weight (%basal). Absolute pre-fasting body weights at 18 weeks of age were WT male  = 25.4±0.6 g; Y1Y5^−/−^ male  = 29.3±1.8 g; WT female  = 19.7±0.5 g; Y1Y5^−/−^ female  = 20.1±0.4 g, and at 34 weeks of age, WT male  = 30.1±1.3 g; Y1Y5^−/−^ male  = 35.5±1.2 g; WT female  = 24.5±0.7 g; Y1Y5^−/−^ female  = 25.0±0.9 g. Plotted values are means ± SEM of 4–21 standard chow-fed mice per group. **P<0.01 or ***P<0.001 for Y1Y5^−/−^ versus WT mice of the same age and sex.

**Figure 3:**
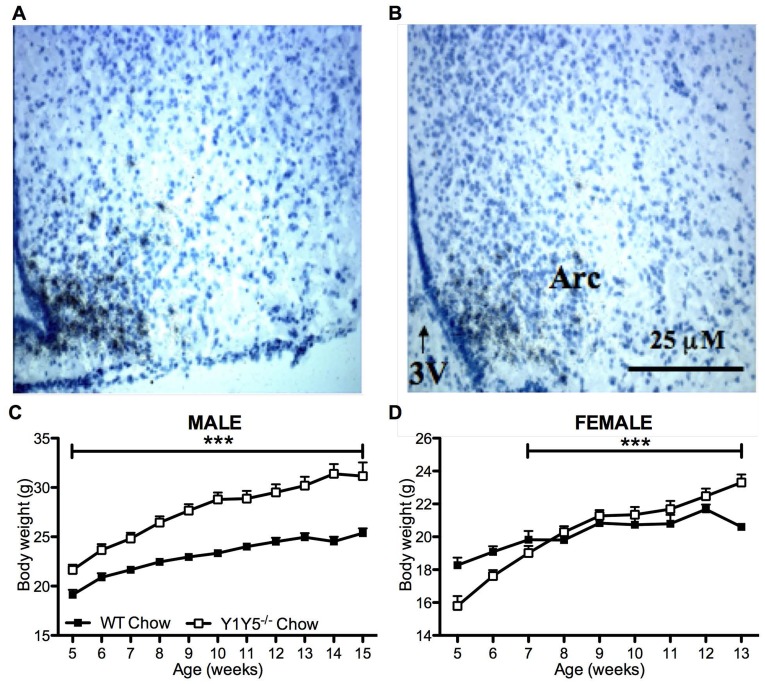
Reduced ARC NPY expression and increased body weight in young Y1Y5^−/−^ mice on chow. (A–B) Bright field photomicrographs of coronal brain sections from 15 week old male wildtype control (WT, A) and germline Y1Y5 receptor knockout (Y1Y5^−/−^, B) mice after *in situ* hybridisation for NPY mRNA. Scale bar  = 25 µm. Abbreviations: 3V; third ventricle, ARC, arcuate nucleus of the hypothalamus. (C–D) Body weight from 5 to 15 weeks of age in male (C) and 5 to 13 weeks of age in female (D) WT or Y1Y5^−/−^ mice. Plotted values are means ± SEM of 4–21 standard chow-fed mice per group. ***P<0.001 for Y1Y5^−/−^ versus WT mice of the same age and sex.

**Table 1:** Hypothalamic neuropeptide expression in male Y1Y5^−/−^ mice on chow.

	WT	Y1Y5^−/−^
ARC NPY mRNA	100±6	68±12*
ARC POMC mRNA	100±4	96±3
PVN TRH mRNA	100±8	88±8

Expression levels of mRNA for neuropeptide Y (NPY), proopiomelanocortin (POMC) and thyrotropin releasing hormone (TRH) as determined by *in situ* hybridisation in the arcuate nucleus of the hypothalamus (ARC) or the hypothalamic paraventricular nucleus (PVN) in male wildtype control (WT) or germline Y1Y5 receptor knockout (Y1Y5^−/−^) mice at 15 weeks of age. mRNA levels are expressed as relative optical density units, relative to that of WT. Data are means ± SEM of 4–6 male mice per group. *P<0.05 versus wildtype mice.

## Methods

### Ethics Statement and Animal Care

All research and animal care procedures were approved by the Garvan Institute/St Vincent’s Hospital Animal Ethics Committee and were in agreement with the Australian Code of Practice for the Care and Use of Animals for Scientific Purposes. All mice were housed under conditions of controlled temperature (22°C) and illumination (12 h light-dark cycle, lights on at 7∶00 hours) with *ad* *libitum* access to water and standard chow (8% calories from fat, 21% calories from protein, 71% calories from carbohydrates and 2.6 kcalories.g^−1^; Gordon’s Specialty Stock Feeds, Yanderra, New South Wales, Australia) unless otherwise stated.

### Generation of Y1Y5 Double Knockout Mice

Due to the close proximity of the Y1 and Y5 genes, being approximately only 20 kb apart [19], generation of double mutant mice is very unlikely to be achieved by simple crossing of the single knockout strains. Therefore a strategy of double targeting of embryonic stem (ES) cells was used. Positively targeted ES cells carrying the mutant Y1 allele were targeted again with a vector containing a Y5 receptor gene flanked by loxP sites (floxed). In order to be able to select for additional targeting events, a different selection marker was used in this Y5 targeting vector (hygromycin), versus the already existing neomycin resistant gene. Standard Southern blot analysis was used to identify clones with the correctly integrated Y5 targeting vector. Long range PCR was then used to verify which of these clones actually had targeting events on the same strand of DNA, as would be necessary for coupled transmission of the mutant genes. Two ES cell lines positive for targeting events of both Y1 and Y5 receptors on the same strand of DNA were then injected into oocytes derived from C57BL/6 mice to generate chimeric offspring and subsequent heterozygous mice carrying the conditionally targeted genes (Y1Y5^lox/+^). Crossing these mice with an oocyte-specific Cre-expressing line, followed by crossing the respective heterozygous offspring, then led to generation of both the germline and conditional Y1 and Y5 double receptor knockout mice, termed Y1Y5^−/−^ and Y1Y5^lox/lox^, respectively. Both of these knockout strategies are designed to result in deletion of the entire coding region of the Y1 and Y5 receptors. All mice were on a mixed C57BL/6-129/SvJ background. Absence of the Y1Y5 genes in homozygous germline Y1Y5^−/−^ mice was confirmed by PCR on genomic DNA as described below and as shown in the results section. The successful deletion of both genes by this cross also provides verification for the functionality of the Cre-lox system, as this targeted locus is important for the conditional deletion of these genes described below.

**Figure 4:**
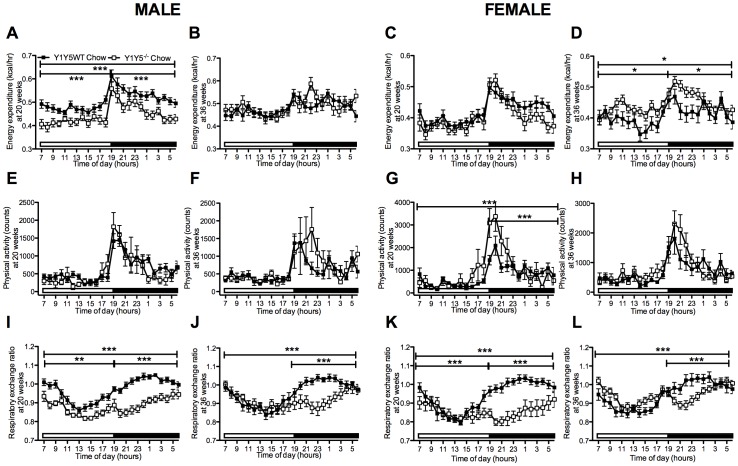
Altered energy expenditure and decreased respiratory exchange ratio in Y1Y5^−/−^ mice on chow. (A–L) 24-hour time course of energy expenditure (A–D), physical activity (E–H) and respiratory exchange ratio (I–L) in male and female germline Y1Y5 receptor knockout (Y1Y5^−/−^) or wildtype control (WT) mice at 20 and 36 weeks of age. Energy expenditure was adjusted for lean mass by analysis of covariance (ANCOVA). Adjusted energy expenditure was presented at a common lean mass of 21.936 g (males) and 16.015 g (females) at 20 weeks of age and 23.172 g (males) and 17.115 g (females) at 36 weeks of age. Open and filled horizontal bars indicate light and dark phases, respectively. Plotted values are means ± SEM of 5–12 standard chow-fed mice per group. *P<0.05, **P<0.01, or ***P<0.001 for Y1Y5^−/−^ versus WT mice of the same age and sex.

### Generation of Adult-onset Hypothalamus-specific Y1Y5 Receptor Knockout Mice

Y1Y5^lox/lox^ mice at 12 weeks of age were anaesthetised with 100/20 mg/kg ketamine/xylazine (Parke Davis-Pfizer, Sydney, Australia and Bayer AG, Leverkusen, Germany). With the head in the flat skull position and using a stereotaxic table (David Kopf, Tujunga, CA, USA), brain injection coordinates relative to Bregma were posterior 0.94 mm, lateral ±0.3 mm, ventral 4.75 mm, corresponding to the paraventricular nucleus (PVN) of the hypothalamus [23]. A volume of 1 µL of viral vector (1 X 10^10^ plaque-forming units per µL) was injected bilaterally over 10 minutes using a 1-µl Hamilton syringe attached to a syringe infusion pump (World Precision Instruments Inc, Walthan, MA, USA). Injection with recombinant adeno-associated viral vector (rAAV) expressing Cre-recombinase was used to produce hypothalamus-specific Y1Y5 receptor knockout mice (Y1Y5^Hyp/Hyp^). As controls, littermate Y1Y5^lox/lox^ mice were injected with a GFP-expressing adeno-associated viral vector.

### High Fat Diet Intervention

At 16 weeks of age and 4 weeks after rAAV vector injection, half of the Y1Y5^Hyp/Hyp^ and Y1Y5^lox/lox^ mice were fed a high fat diet (23% calories from fat, 19.4% calories from protein, 48.2% calories from carbohydrate, 4.7% calories from crude fibre, 4.7% calories from acid detergent fibre, 4.78 kcalories.g^−1^; Gordon’s Specialty Feeds, Glen Forrest, WA, Australia) for 20 weeks. The remaining mice continued on the standard chow diet.

### Feeding Studies

Food intake was measured both in the fed state (referred to as spontaneous food intake), as well as in response to 24-hour fasting using Nalgene metabolic cages (Medtex, Notting Hill, VIC). Mice were transferred to individual cages and were fed a powdered diet for 3 days before being placed into individual Nalgene metabolic cages. Spontaneous food intake was determined as the average of triplicate readings taken over three consecutive 24-hour periods. Twenty four-hour fasting-induced food intake was subsequently measured at 1, 2, 4, 7, 24 and 48 hours after re-introduction of food to the cages, and body weight was tracked throughout feeding studies.

**Figure 5:**
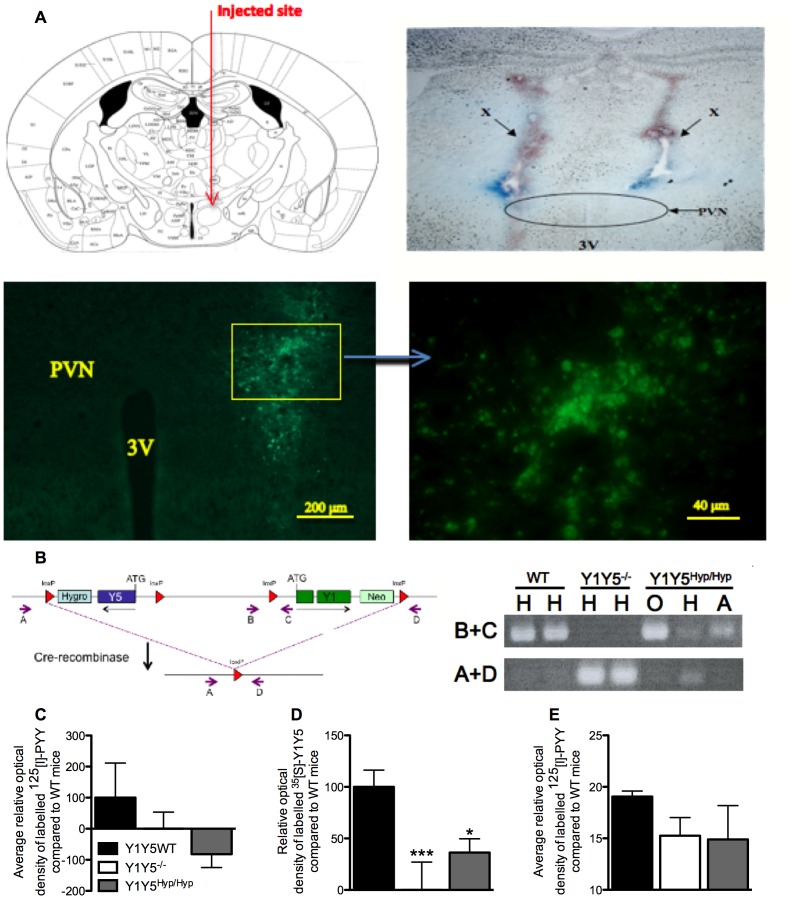
Successful deletion of hypothalamic Y1Y5 receptors from adult mice. (A) Top left: Schematic diagram of proposed injection site just outside the PVN (adapted with permission from [23]). Top right: Coronal brain section showing the position of needles used to inject Cre-recombinase- or GFP-expressing viral vector into the brain, as shown by the Bromophenol blue-stained needle tracks at X. By targeting delivery to just outside the PVN, the nucleus is undamaged by the needle tip but viral vector can diffuse through it. Bottom left: Coronal brain section demonstrating the localised expression (indicated by fluorescence) of the GFP-expressing viral vector targeted to just outside of the PVN. Bottom right: Localised fluorescence from the image at bottom left seen at higher magnification. (B) Targeting vector design illustrating loxP sites flanking the Y1 and Y5 receptor genes. Small arrows indicate the position and orientation of oligonucleotides used in PCR analysis. Inset: PCR analysis of genomic DNA isolated from sections of the hypothalamus containing the PVN (H), olfactory bulb (O) or the arcuate nucleus (A) of two wildtype (WT), two germline Y1Y5 receptor knockout (Y1Y5^−/−^) mice, or a conditional knockout Y1Y5^lox/lox^ mouse injected with a Cre-recombinase-expressing viral vector into the PVN (Y1Y5^Hyp/Hyp^). When oligonucleotides B+C are used, a PCR product is clearly produced in DNA isolated from the PVN (H) of WT mice, and in DNA isolated from the olfactory bulb (O) or arcuate nucleus (A) from Y1Y5^Hyp/Hyp^ mice, demonstrating the presence of intact Y1Y5 receptors in both brain regions in these mice. In contrast, when oligonucleotides A+D are used, a PCR product is produced only from DNA isolated from sections of the hypothalamus containing the PVN (H) of Y1Y5^−/−^ and Y1Y5^Hyp/Hyp^ mice, and not from WT mice or from sections of the hypothalamus containing the olfactory bulb (O) or the arcuate nucleus (A) of Y1Y5^Hyp/Hyp^ mice, demonstrating successful deletion of the Y1 and Y5 genes specifically from the targeted PVN region. (C) ^125^[I]-peptide YY (PYY) binding levels in the PVN of wildtype (Y1Y5WT), Y1Y5^−/−^ or Y1Y5^Hyp/Hyp^ mice. (D) Y1Y5 mRNA expression in the PVN of Y1Y5WT, Y1Y5^−/−^ and Y1Y5^Hyp/Hyp^ mice after subtraction of Y1Y5^−/−^ values as a background control. (E) ^125^[I]-PYY binding levels in the arcuate nucleus of Y1Y5WT, Y1Y5^−/−^ and Y1Y5^Hyp/Hyp^ mice. Plotted values are means ± SEM of the averages of 2–7 mice per group, with 3–9 separate measurements per mouse. *P<0.05, ***P<0.001 versus WT mice. Abbreviations: 3V, third ventricle; PVN, hypothalamic paraventricular nucleus.

**Figure 6:**
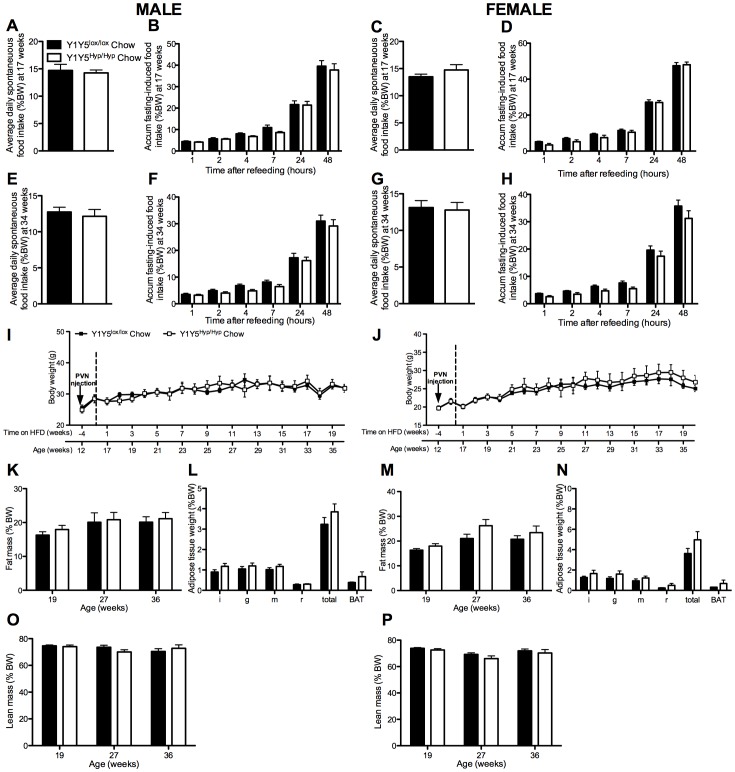
No effect of adult-onset hypothalamus-specific Y1Y5 receptor deletion on energy homeostasis on a chow diet. (A–H) Spontaneous (A, C, E, G) and accumulated (accum) 24-hour fasting-induced food intake (B, D, F, H), normalised to body weight, in adult-onset hypothalamus-specific Y1Y5 receptor deficient (Y1Y5^Hyp/Hyp^) or control (Y1Y5^lox/lox^) mice at 18 and 34 weeks of age. (I–J) Body weight from 12 to 36 weeks of age in male (I) and female (J) Y1Y5^Hyp/Hyp^ or Y1Y5^lox/lox^ control mice. ‘PVN injection’ refers to the time of induction of gene deletion by injection of a Cre-recombinase-expressing viral vector into the paraventricular nucleus of the hypothalamus. (K–P) Fat and lean masses as a percentage of body weight (%BW), measured using dual energy x-ray absorptiometry, in male (K, O) and female (M, P) Y1Y5^Hyp/Hyp^ and Y1Y5^lox/lox^ mice at 19, 27 and 36 weeks of age. Weight (as %BW) of dissected white (WAT) and brown adipose tissue (BAT) depots at the end of the study in male (L) and female (N) Y1Y5^Hyp/Hyp^ and Y1Y5^lox/lox^ mice at 36 weeks of age. Abbreviations: i, inguinal; g, gonadal; m, mesenteric; r, retroperitoneal; total, summed weight of i, g, m and r WAT depots. Plotted values are means ± SEM of 5–12 standard chow-fed mice per group.

**Figure 7:**
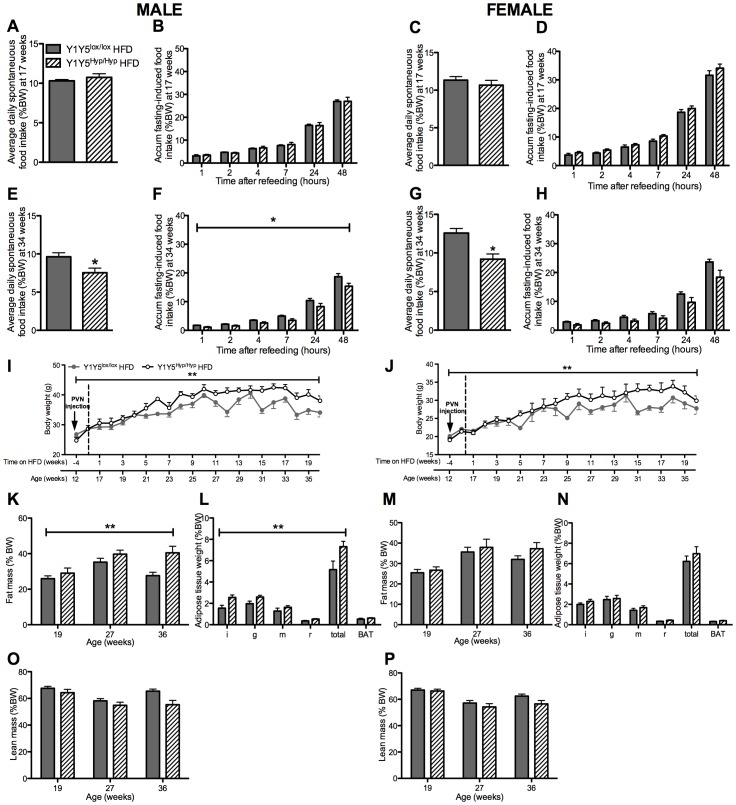
Obesity despite hypophagia after adult-onset hypothalamus-specific Y1Y5 deletion in mice on a high fat diet. (A–H) Spontaneous (A, C, E, G) and accumulated (accum) 24-hour fasting-induced food intake (B, D, F, H), normalised to body weight, in adult-onset hypothalamus-specific Y1Y5 receptor deficient (Y1Y5^Hyp/Hyp^) or control (Y1Y5^lox/lox^) mice at 18 and 34 weeks of age. (I–J) Body weight from 12 to 36 weeks of age in male (I) and female Y1Y5^Hyp/Hyp^ or Y1Y5^lox/lox^ mice. Mice were on the high fat diet (HFD) from 16 weeks of age onwards. ‘PVN injection’ refers to the time of induction of gene deletion by injection of a Cre-recombinase-expressing viral vector into the paraventricular nucleus of the hypothalamus. (K–P) Fat and lean masses as a percentage of body weight (%BW), measured using dual energy x-ray absorptiometry, in male (K, O) and female (M, P) Y1Y5^Hyp/Hyp^ and Y1Y5^lox/lox^ mice at 19, 27 and 36 weeks of age. Weight (as %BW) of dissected white (WAT) and brown adipose tissue (BAT) depots at the end of the study in male (L) and female (N) Y1Y5^Hyp/Hyp^ and Y1Y5^lox/lox^ mice at 36 weeks of age. Abbreviations: i, inguinal; g, gonadal; m, mesenteric; r, retroperitoneal; total, summed weight of i, g, m and r WAT depots. Plotted values are means ± SEM of 5–12 HFD-fed mice per group. *P<0.05 or **P<0.01 for Y1Y5^Hyp/Hyp^ versus Y1Y5^lox/lox^ mice of the same age and sex.

### Glucose Metabolism in Hypothalamus-specific Y1Y5 Receptor Knockout Mice

At 18 and 35 weeks of age, corresponding to 6 and 23 weeks after hypothalamic rAAV injection and 2 and 19 weeks on a high fat diet, Y1Y5^Hyp/Hyp^ and Y1Y5^lox/lox^ control mice underwent an insulin tolerance test at 14∶00 to 16∶00 hours. Briefly, the mice were fasted for 6 hours and then intraperitoneally injected with insulin (0.5 IU/kg) (Novo Nordisk Pharmaceuticals, Baulkham Hills, NSW, Australia). Tail tip blood samples were then collected at 0, 15, 30, 45, 60, 75 and 90 minutes after insulin injection, and blood glucose levels were measured using a glucometer (AccuCheck II; Roche, New South Wales, Castle Hill, Australia). Four days later, mice were fasted for 16 to 24 hours before intraperitoneal injection between 13∶00 to 15∶00 hours of a 10% D-glucose solution (1.0 g/kg) (Astra Zeneca, North Ryde, NSW, Australia). Blood samples were obtained from the tail tip at 0, 15, 30, 60 and 90 minutes after glucose injection and blood glucose levels were measured using a glucometer.

### Indirect Calorimetry

Energy expenditure and respiratory exchange ratio were calculated by measurement of the rate of oxygen consumption (VO_2_) and carbon dioxide output (VCO_2_) via indirect calorimetry in *ad libitum*-fed mice using an eight-chamber open-circuit calorimeter (Oxymax Series; Columbus Instruments, Columbus, OH, USA) as previously described by us [16]. Physical activity was also determined in the calorimetry cages using an OPTO-M3 sensor system (Columbus Instruments) as previously described [16]. Studies were conducted after 24 hours of acclimatisation to calorimetry chambers.

**Figure 8:**
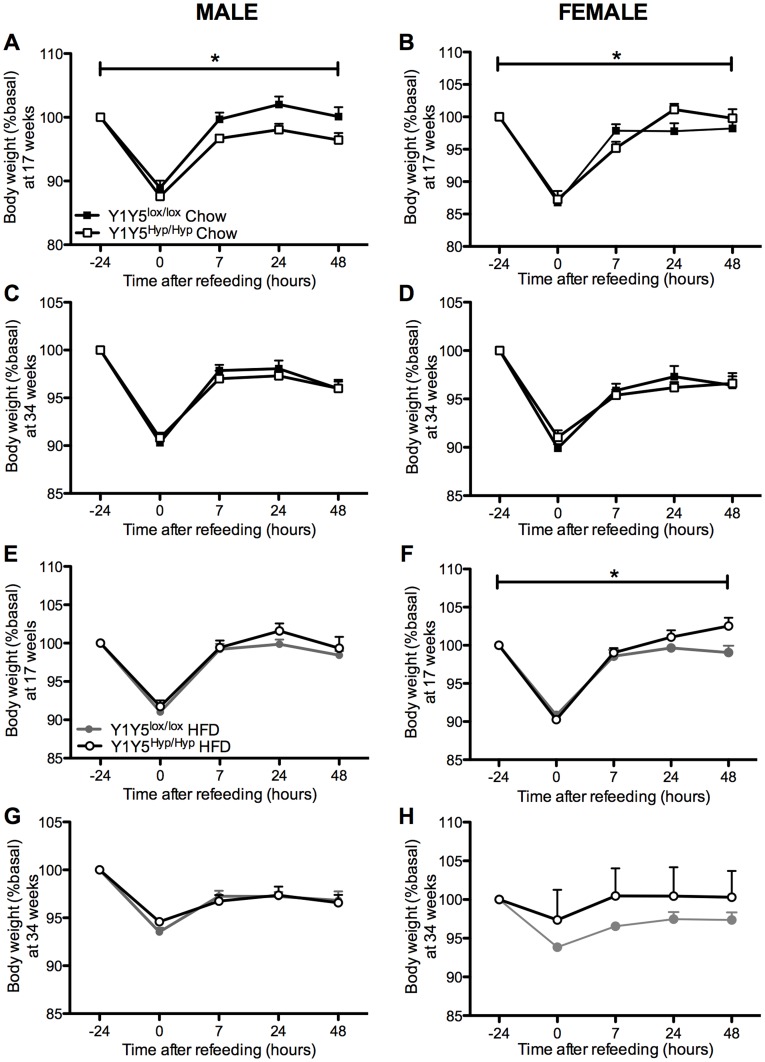
Effect of adult-onset hypothalamus-specific Y1Y5 receptor deletion on weight regain after a 24-hour fast. Body weight measurements were taken at the time points of fasting-induced food intake measurements in male (A, C, E, G) and female (B, D, F, H) adult-onset hypothalamus-specific Y1Y5 receptor knockout (Y1Y5^Hyp/Hyp^) or control (Y1Y5^lox/lox^) mice, at both 17 and 34 weeks of age and on a standard chow (Chow) or a high fat diet (HFD). Data are represented as a percent of pre-fasting body weight (%basal). Absolute pre-fasting body weights at 17 weeks of age were Y1Y5^lox/lox^ male  = 25.4±0.6 g (standard chow), 28.0±0.6 g (HFD); Y1Y5^−/−^ male  = 26.1±0.9 g (standard chow), 28.9±0.9 g (HFD); Y1Y5^lox/lox^ female  = 20.1±0.5 g (standard chow), 21.6±1.0 g (HFD); Y1Y5^−/−^ female  = 20.1±0.6 g (standard chow), 21.6±0.8 g (HFD), and at 34 weeks of age were Y1Y5^lox/lox^ male  = 30.0±1.3 g (standard chow), 34.0±1.5 g (HFD); Y1Y5^−/−^ male  = 31.2±1.6 g (standard chow), 40.2±1.6 g (HFD); Y1Y5^lox/lox^ female  = 24.7±0.7 g (standard chow), 27.9±1.5 g (HFD); Y1Y5^−/−^ female  = 27.1±2.0 g (standard chow), 29.9±1.3 g (HFD). Plotted values are means ± SEM of 4–21 mice per group. *P<0.05 for Y1Y5^Hyp/Hyp^ versus Y1Y5^lox/lox^ mice of the same age, sex and diet.

### Determination of Body Composition

Upon completion of indirect calorimetry and physical activity measurements, animals were anesthetised with isoflurane and then scanned (with the head excluded and the tail included) using dual-energy X-ray absorptiometry (DXA, Lunar PIXImus2 mouse densitometer; GE Healthcare, Waukesha, WI, USA) to determine whole body fat and lean mass.

### Tissue Collection

At 36 weeks of age, mice were fasted from 09∶00 hours and culled between 13∶00 to 17∶00 hours by cervical dislocation followed by decapitation. The brain was removed and frozen on an aluminium plate on dry ice, then stored at −80°C until analyses as described below. The interscapular brown adipose tissue (BAT) as well as white adipose tissue (WAT) depots (right inguinal, right epididymal or periovarian (gonadal), right retroperitoneal and mesenteric) were removed and weighed. The weights of these WAT depots were summed together and expressed as total WAT weight, normalised as a percent of body weight.

### Polymerase Chain Reaction Confirmation of Germline and Hypothalamus-specific Y1Y5 Receptor Deletion in Y1Y5^−/−^ and Y1Y5^Hyp/Hyp^ Mice

Y1 and Y5 receptor deletion was confirmed using genomic DNA isolated from various brain regions of Y1Y5^−/−^ or Y1Y5^Hyp/Hyp^ mice. The brain tissue used for DNA extraction was dissected from one of three regions: a section including the PVN, a section including the olfactory bulb, and a section including the hypothalamic arcuate nucleus. PCR was performed with the following primers: Oligo A, 5′-CCATGAAGCATGTTGTGG-3′; Oligo B, 5′-ATTCTCTAGTCAAAGTGTC-3′; Oligo C, 5′-CTTGTAGCAAATTCCTGCAG-3′; and Oligo D, 5′-GCCAGAGATAGATTACAAAG-3′. PCR with the combination of Oligos B and C produces a 314 bp fragment representing the homozygous floxed Y1Y5^lox/lox^ genotype. Combination A and D produces a product of 150 bp only when the entire Y1Y5 gene cluster has been removed, confirming successful deletion of both the Y1 and Y5 receptor gene.

**Figure 9:**
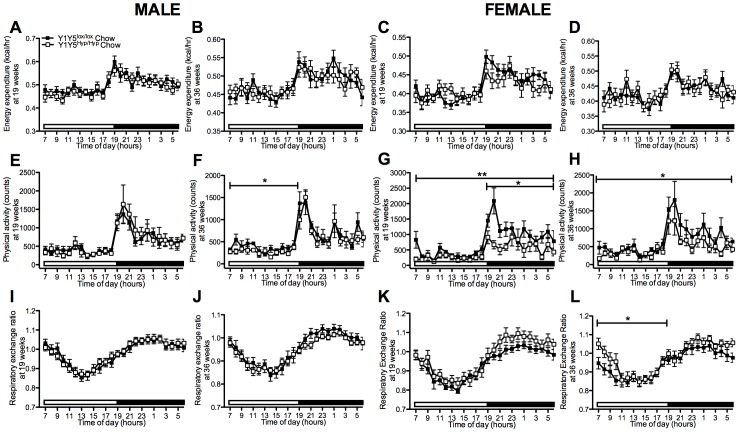
Unaltered energy expenditure and decreased physical activity in chow-fed mice with adult-onset hypothalamus-specific Y1Y5 receptor deletion. (A–L) 24-hour time course of energy expenditure (A–D), physical activity (E–H) and respiratory exchange ratio (I–L) in male and female adult-onset hypothalamus-specific Y1Y5 receptor knockout (Y1Y5^Hyp/Hyp^) or control (Y1Y5^lox/lox^) mice at 19 and 36 weeks of age. Energy expenditure was adjusted for lean mass by analysis of covariance (ANCOVA). Adjusted energy expenditure was presented at a common lean mass of 21.629 g (males) and 16.252 g (females) at 19 weeks of age and 22.762 g (males) and 18.865 g (females) at 36 weeks of age. Open and filled horizontal bars indicate light and dark phases, respectively. Plotted values are means ± SEM of 5–12 standard chow-fed mice per group. *P<0.05 or **P<0.01 for Y1Y5^Hyp/Hyp^ versus Y1Y5^lox/lox^ mice of the same age and sex.

**Figure 10:**
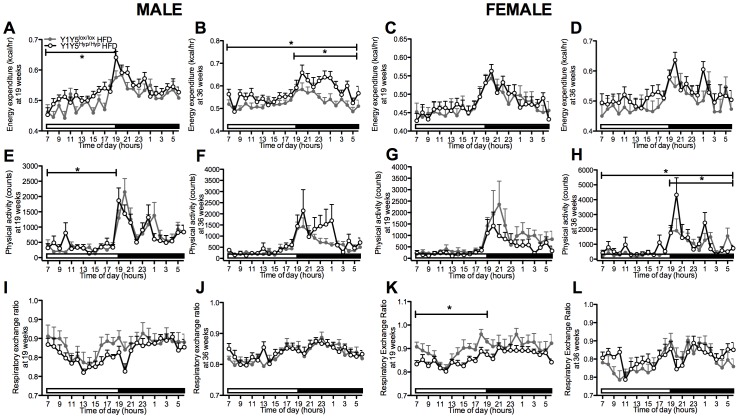
Increased energy expenditure/physical activity in high fat-fed mice with adult-onset hypothalamus-specific Y1Y5 receptor deletion. (A–L) 24-hour time course of energy expenditure (A–D), physical activity (E–H) and respiratory exchange ratio (I–L) in male and female adult-onset hypothalamus-specific Y1Y5 receptor knockout (Y1Y5^Hyp/Hyp^) or control (Y1Y5^lox/lox^) mice on a high fat diet (HFD) at 19 and 36 weeks of age. Energy expenditure was adjusted for lean mass by analysis of covariance (ANCOVA). Adjusted energy expenditure was presented at a common lean mass of 20.741 g (males) and 15.607 g (females) at 19 weeks of age and 21.636 g (males) and 17.320 g (females) at 36 weeks of age. Open and filled horizontal bars indicate light and dark phases, respectively. Plotted values are means ± SEM of 5–12 HFD-fed mice per group. *P<0.05 or ***P<0.001 for Y1Y5^Hyp/Hyp^ versus Y1Y5^lox/lox^ mice of the same age and sex.

### Radioactive Ligand Binding Assay for Confirmation of Successful Hypothalamus-specific Y1Y5 Receptor Deletion and Determination of Total Y Receptor Binding

Coronal brain sections, 25 µm thick at the level of the PVN or hypothalamic arcuate nucleus, were cut and thaw-mounted on charged slides and stored at −20°C until radioactive ligand binding assay as previously described [24]. In brief, sections were incubated with 25 pM [^125^I]-PYY to demonstrate total Y receptor binding, or with 25 pM [^125^I]-PYY with or without addition of 1 µM of the Y_2_ receptor antagonist BIIE0246, indicating the degree to which Y2-receptor binding is altered.

### *In Situ* Hybridisation for Quantification of Hypothalamic NPY, POMC, TRH and Y1 and Y5 Receptor mRNA Expression

Slides with frozen brain sections containing the arcuate nucleus or PVN were thawed and hybridised with radiolabelled oligonucleotides for quantification of mRNA levels as previously described [25].

### Statistical Analyses

All data are expressed as means ± SEM. Differences between knockout and control mice were assessed by ANOVA or repeated measures ANOVA, with Fisher’s post hoc tests where appropriate. Energy expenditure, respiratory exchange ratio and physical activity over the continuous 24-hour period were averaged for the whole 24-hour period, as well as for the light and dark periods. Comparison of energy expenditure (kcal/hr) was carried out by analysis of covariance (ANCOVA) with lean mass as a covariate. Adjusted means of energy expenditure at a common lean mass were generated by ANCOVA and presented. Statistical analyses were performed with SPSS for Mac OS X version 16.0.1 (SPSS Inc, Chicago, IL, USA). Statistical significance was defined as P<0.05.

**Figure 11:**
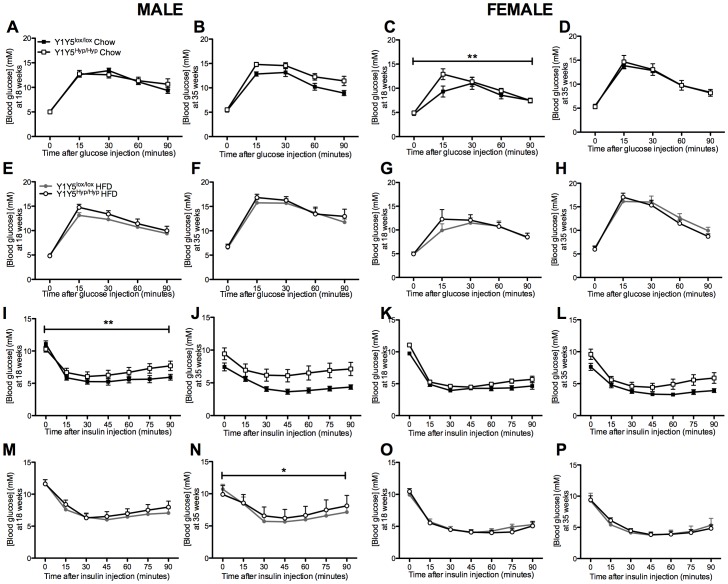
Altered blood glucose responses to glucose and insulin after adult-onset hypothalamus-specific Y1Y5 receptor deletion. (A–H) Intraperitoneal glucose tolerance tests (1 g/kg) were conducted in 24-hour fasted male (A, B, E, F) and female (C, D, G, H) adult-onset hypothalamus-specific Y1Y5 receptor deficient (Y1Y5^Hyp/Hyp^) or control (Y1Y5^lox/lox^) mice at 18 or 35 weeks of age. (I–P). Intraperitoneal insulin tolerance tests (0.5 U/kg) were conducted in 6-hour fasted male (I, J, M, N) and female (K, L, O, P) Y1Y5^Hyp/Hyp^ or control Y1Y5^lox/lox^ mice at 18 or 35 weeks of age. Plotted values are means ± SEM of 5–12 chow or high fat diet (HFD)-fed mice per group. *P<0.05 or **P<0.01 for Y1Y5^Hyp/Hyp^ versus Y1Y5^lox/lox^ mice of the same age, sex and diet.

## Results

### Absence of Y1 and Y5 Signalling Reduces Spontaneous and Fasting-induced Food Intake

Since pharmacological evidence suggests that both Y1 and Y5 receptors play a critical role in the regulation of food intake [10]–[12], we first investigated spontaneous and fasting-induced food intake in germline Y1Y5 double knockout mice. Importantly, male Y1Y5^−/−^ mice at both 18 and 34 weeks of age showed significantly decreased spontaneous food intake (Fig. 1A, E). There was no such decrease in female Y1Y5^−/−^ mice (Fig. 1C, G). As hypothalamic NPY expression and Y receptor activation is enhanced under fasting conditions [26], [27], we hypothesised that any differences in food intake between genotypes would be more apparent after fasting. Indeed, both, male and female Y1Y5^−/−^ mice at 18 weeks of age, and male Y1Y5^−/−^ mice at 34 weeks of age, showed significant reductions in food intake for up to 48 hours after a 24-hour fast (Fig. 1B, D, F, H). This reduction in food intake translated into delayed body weight recovery after fasting in Y1Y5^−/−^ versus wildtype mice (Fig. 2). Although male and female Y1Y5^−/−^ animals at 18 weeks of age lost a similar percent body weight to wildtypes during the 24-hour fast, they exhibited significantly reduced body weight – as a percent of pre-fasting values – during the 48 hours of refeeding (Fig. 2A, B). This delayed weight regain after fasting was also seen in male, but not female Y1Y5^−/−^ mice at 34 weeks of age (Fig. 2C, D). Furthermore, the decreased food intake of Y1Y5^−/−^ mice was associated with a decrease in hypothalamic NPY mRNA expression in the arcuate hypothalamic nucleus (ARC) (Fig. 3A, B; Table 1). Interestingly, the mRNA expression of another known major hypothalamic regulator of food intake – proopiomelanocortin (POMC) – was unaltered in the ARC of Y1Y5^−/−^ mice compared to wildtype controls (Table 1), suggesting that lack of NPY signalling through Y1 and Y5 receptors is sufficient to reduce food intake without any concomitant increase in POMC production.

Interestingly, despite their significantly reduced food intake, accelerated weight gain was observed in Y1Y5^−/−^ mice from 5 to 15 weeks of age, particularly in males (Fig. 3C, D). Weekly body weight measurements in a separate cohort of older mice, from 16 to 36 weeks of age, show that male but not female Y1Y5^−/−^ mice exhibited significantly increased body weight compared to wildtype mice (Fig. 1I, J). Dual energy X ray absorptiometry (DXA) revealed increases in fat mass as a percent of body weight when measured at 20 and 36 weeks of age in Y1Y5^−/−^ mice, significantly so in females (Fig. 1K, M). This was confirmed by regional white adipose tissue (WAT) dissection at 36 weeks of age (Fig. 1L, N). Female but not male Y1Y5^−/−^ animals demonstrated a significant decrease in lean mass at 20 and 36 weeks of age compared to wildtype counterparts, as determined with DXA analysis (Fig. 1O, P). This may have contributed to the lack of increase in body weight in older female Y1Y5^−/−^ mice despite increased adiposity.

### Energy Expenditure and Respiratory Exchange Ratio are Reduced in Germline Y1Y5^−/−^ Mice

In order to investigate the discrepancy between reduced food intake and elevated body weight gain we used indirect calorimetry to determine whether differences in energy metabolism may have contributed to the increased body weight or adiposity of Y1Y5^−/−^ mice despite reduced food intake. Energy expenditure was reduced in Y1Y5^−/−^ compared to wildtype mice at 20 weeks of age, and this difference was statistically significant in males (Fig. 4A, C). Conversely, energy expenditure was significantly increased in female Y1Y5^−/−^ versus wildtype mice at 36 weeks of age (Fig. 4D). This effect was not seen in male double mutant mice of the same age (Fig. 4B). These changes in energy expenditure were not due to corresponding reductions in physical activity in Y1Y5^−/−^ mice of either sex or either age (Fig. 4E–H). In fact, physical activity was significantly increased relative to wildtype values in female Y1Y5^−/−^ mice at 20 weeks of age (Fig. 4G). Respiratory exchange ratio (RER), an index of fuel source, was significantly decreased in both male and female Y1Y5^−/−^ compared to wildtype mice at both 20 and 36 weeks of age, particularly during the dark phase (Fig. 4I–L), suggesting a propensity for Y1Y5^−/−^ mice to utilise more fat as their oxidative fuel source compared to wildtype mice. It should be noted that the RER data in our studies, at times, exceeds a value of 1, which is likely a reflection of lipogenesis taking place at that point in time [28], [29]. Taken together, these data show that Y1Y5 receptor deletion in mice, especially males, results in greater body weight and adiposity which occurs despite reduced spontaneous or fasting-induced food intake, as well as a greater propensity to oxidise fat as a fuel source. A possible contributor to this increased weight gain and adiposity in Y1Y5^−/−^ mice, at least in males, is the decreased energy expenditure seen at the earlier age of 20 weeks.

### Adult-onset Hypothalamus-specific Y1Y5 Receptor Dual Ablation Reduces Food Intake

In order to determine whether hypothalamic Y1 and Y5 receptors are the critical components for the hypophagic effect of germline double knockout, we studied adult-onset hypothalamus-specific Y1Y5 deleted mice. For this, Y1Y5^lox/lox^ mice were injected into the hypothalamic paraventricular nucleus (PVN) region with a recombinant Cre-recombinase-expressing adeno-associated viral vector (rAAV). These mice are referred to as Y1Y5^Hyp/Hyp^. Control mice were injected with a GFP-expressing AAV. Delivery was targeted to just above the PVN to avoid destruction of the nucleus, and was close enough to allow diffusion of the rAAV-Cre virus into the PVN, as can be seen in Fig. 5A from the position of the needle track and of the localised fluorescence from viral vector-derived GFP in this nucleus. Hypothalamic gene deletion was confirmed using PCR amplification of Y1 and Y5 receptors (Fig. 5B), demonstrating that Y1Y5 receptor deletion was specific to parts of the hypothalamus containing the PVN, with no gene deletion occurring in parts of the hypothalamus containing the olfactory bulb or the arcuate nucleus, an area in close proximity to the targeted injection site (the PVN). Radioactive ligand binding using ^125^[I]-peptide YY (PYY) to detect total Y receptor binding (Fig. 5C), as well as *in situ* hybridisation with oligonucleotides to detect Y1 and Y5 receptor mRNA (Fig. 5D), demonstrate reduced total Y receptor binding and reduced Y1 and Y5 mRNA expression in the PVN of Y1Y5^−/−^ and Y1Y5^Hyp/Hyp^ mice relative to wildtype controls, this change being statistically significant for Y1Y5 mRNA levels (Fig. 5D). As Y1 and Y5 receptors are the predominant Y receptors expressed in the PVN [30], these changes in total Y receptor binding and Y1Y5 mRNA expression in the PVN thereby confirm effective Y1Y5 receptor gene knockdown in the hypothalamus of Y1Y5^Hyp/Hyp^ mice. To investigate possible effects of Y1Y5 receptor deletion on the expression of other Y receptors in a key brain region regulating energy homeostasis, we measured ^125^[I]-PYY binding in the arcuate nucleus of our mouse models, providing an index of total Y receptor binding in this region. We also specifically investigated changes in Y2 receptor binding in the arcuate nucleus of our mice by determining the degree to which ^125^[I]-PYY binding was displaced by the Y2-preferring antagonist, BIIE0246. We did not specifically investigate Y4 or y6 binding because Y4 receptors represent only a very small percentage of Y receptors in the brain and specifically in the hypothalamus [30], and because y6 receptors are only found in the suprachiasmatic nucleus [31]. However, neither of our Y1Y5 deficient models demonstrated significant differences from wildtype mice with respect to total Y receptor binding (Fig. 5E) nor Y2 receptor binding (data not shown) in the arcuate nucleus.

At 16 weeks of age, corresponding to 4 weeks after stereotaxic brain injection, half of the Y1Y5^lox/lox^ and Y1Y5^Hyp/Hyp^ animals were placed on a high fat diet (HFD) for 20 weeks. The remaining mice were continued on the standard chow diet. We investigated all mice at 1–3 and 18–20 weeks after commencement of the HFD, corresponding to 17–19 and 34–36 weeks of age, respectively. On the standard chow diet, Y1Y5^Hyp/Hyp^ mice exhibited no significant differences in food intake, albeit there was a trend to reduced fasting-induced food intake (Fig. 6A–H). When fed the HFD, however, male and female mice with hypothalamic Y1Y5 receptor deficiency exhibited a significantly reduced spontaneous and/or fasting-induced food intake when investigated at 34 (Fig. 7E–H) but not at 17 weeks of age (Fig. 7A–D).

As in the Y1Y5^−/−^ mice, 17 week-old male Y1Y5^Hyp/Hyp^ mice on a standard chow diet lost a similar proportion of their body weight in response to fasting as wildtypes, and exhibited delayed body weight recovery during refeeding (Fig. 8A), albeit this effect was not seen in chow-fed male mice at the later time point of 34 weeks, nor in high fat-fed males, nor in female Y1Y5^Hyp/Hyp^ mice on either diet or at either time point (Fig. 8B-H). In fact, female Y1Y5^Hyp/Hyp^ mice on either diet exhibited significantly faster body weight recovery than their wildtype counterparts (Fig. 8B, F).

Unlike the Y1Y5^−/−^ mice, the body weight, adiposity and lean mass of male and female Y1Y5^Hyp/Hyp^ mice on the standard chow diet was not statistically different from corresponding measures in Y1Y5^lox/lox^ controls (Fig. 6I–P). In contrast, and despite concomitant hypophagia, the body weight of male and female Y1Y5^Hyp/Hyp^ mice on a HFD was significantly greater than that of Y1Y5^lox/lox^ controls (Fig. 7I, J). Male but not female Y1Y5^Hyp/Hyp^ mice on a HFD exhibited a significant increase in percent fat mass as determined by DXA (Fig. 7K, M), as well as a significant increase in the relative weight of dissected WAT depots relative to control animals (Fig. 7L, N). Lean mass was no different in Y1Y5^Hyp/Hyp^ mice compared to controls at 19, 27 or 36 weeks of age (Fig. 7O, P).

### Altered Energy Expenditure and Physical Activity in Hypothalamus-specific Y1Y5 Receptor Knockout Mice

On the normal chow diet, Y1Y5^Hyp/Hyp^ mice showed no significant difference in energy expenditure relative to wildtype values (Fig. 9A–D). Physical activity was decreased in male and female Y1Y5^Hyp/Hyp^ versus control mice on the standard chow diet at either 19 and/or 36 weeks of age (Fig. 9E–H). On the HFD, energy expenditure was significantly increased in male but not female Y1Y5^Hyp/Hyp^ relative to Y1Y5^lox/lox^ control mice at both 19 weeks of age, after 3 weeks on the HFD, as well as at 36 weeks of age, after 20 weeks on the HFD (Fig. 10A–D). These changes in energy expenditure were associated with corresponding increases in physical activity in male Y1Y5^Hyp/Hyp^ mice at 19 weeks of age, as well as in male and female Y1Y5^Hyp/Hyp^ mice at 36 weeks of age, significantly so in females (Fig. 10E–H). Unlike germline Y1Y5^−/−^ mice, which showed significant decreases in RER, there was no general significant difference between Y1Y5^Hyp/Hyp^ and control mice on the chow diet or HFD with respect to RER, apart from a similar significant decrease in RER during the light phase in HFD-fed female Y1Y5^Hyp/Hyp^ mice at 19 weeks of age, as well as a small but significant increase in RER in chow-fed female knockouts at 36 weeks of age (Fig. 9I–L, Fig. 10I–L). These differences between germline and hypothalamus-specific Y1Y5 receptor knockout mice with respect to RER suggest that it is not the hypothalamic Y1Y5 receptors that control fuel oxidation source, but this is instead controlled by Y1Y5 receptors located outside of this area.

### Involvement of Hypothalamic Y1Y5 Receptors in Regulation of Glucose Metabolism

Since overweight or obese individuals often exhibit glucose intolerance and signs of insulin resistance, and as hypothalamic Y1 and Y5 receptors are implicated in the regulation of glucose homeostasis [32], we measured several parameters of glucose metabolism in Y1Y5^Hyp/Hyp^ mice. After intraperitoneal glucose injection, female but not male Y1Y5^Hyp/Hyp^ mice on a standard chow diet at 18 but not 35 weeks of age demonstrated significantly higher serum glucose levels (Fig. 11A–D). No such genotype effect was observed in mice on the HFD (Fig. 11E–H). After intraperitoneal insulin injection, male Y1Y5^Hyp/Hyp^ mice displayed a significantly blunted drop in serum glucose levels, and this difference was significant at 18 weeks of age on the standard chow diet (Fig. 11I) and at 35 weeks old on the HFD (Fig. 11N). No such effect of hypothalamic Y1Y5 dual deletion was seen in female mice (Fig. 11K, L, O, P).

## Discussion

This study for the first time conclusively demonstrates that food intake in mice requires the coordinated action of both the Y1 and the Y5 receptors, since the simultaneous ablation of both receptors in germline Y1Y5 receptor double knockout (Y1Y5^−/−^) mice lead to reductions in spontaneous and/or fasting-induced food intake at 18 or 34 weeks of age, as well as corresponding delays in weight regain after fasting. This hypophagic effect of germline Y1Y5 ablation is at least partially mediated in the hypothalamus, because mice with adult-onset hypothalamus-specific Y1Y5 receptor dual ablation also exhibited reduced fasting-induced and/or spontaneous food intake at 34 weeks of age and under conditions of a high fat diet (HFD). Despite hypophagia, Y1Y5 deficient mice exhibited increased body weight and/or increased adiposity. This obesity was more apparent in germline than in adult-onset hypothalamus-specific Y1Y5 deficient mice – the latter model exhibiting obesity only on a HFD and not on normal chow – possibly due to compensatory responses to gene deletion such as the decrease in energy expenditure observed in male Y1Y5^−/−^ mice. Taken together, these data reveal the coordinated role of Y1 and Y5 receptors in the physiological regulation of food intake in mice.

The strong hypophagic phenotype of our Y1Y5 double knockout models suggests that Y1 and Y5 receptors play redundant roles in the regulation of food intake. Whereas single germline deletion of Y1 receptors in male and female mice has been consistently shown to reduce fasting-induced but not spontaneous food intake [13]–[16], and whereas single germline deletion of Y5 receptors actually *increases* spontaneous and fasting-induced food intake in both sexes [17], [18], our male germline Y1Y5 receptor double knockout mice exhibited hypophagia under both fasted and non-fasted conditions. These findings imply that when either the Y1 or the Y5 receptor is missing, the remaining receptor is able to compensate or overcompensate to protect against hypophagia. When both receptors are missing, hypophagia ensues, significantly so in males.

It is noteworthy that the hypophagic effect of dual Y1Y5 receptor deletion was more marked in germline than in adult-onset hypothalamus-specific knockout mice. Indeed, the latter model only demonstrated significant reductions in spontaneous or fasting-induced food intake on a HFD but not when fed normal chow, and this effect was only observed at 22 but not at 5 weeks after induction of gene deletion, corresponding to 18 and 1 week on the HFD, respectively. One possible explanation for this discrepancy between models is that the rAAV-derived Cre-recombinase deleted Y1 and Y5 receptors only from a proportion of the PVN neurons targeted in this study. While brain injection of Cre-recombinase-producing adenoviral vectors into conditional knockout mice has been shown to induce gene deletion in up to 90% of neurons [33], we cannot exclude the possibility that our bilaterally-injected rAAV expressing Cre-recombinase did not reach all neurons of the PVN. Also, other areas of the brain besides the PVN are involved in the regulation of feeding, particularly under non-fasted conditions. For instance, NPY-ergic projections to the dorsomedial nucleus of the hypothalamus (DMN) and the ventromedial hypothalamus (VMH) have been implicated in appetite regulation [34], and both of these nuclei are known to express Y1 and/or Y5 receptor mRNA [35], [36]. Studies targeting Y1Y5 receptor deletion in regions such as the DMN and VMH would shed light on this issue.

While we have shown that dual Y1 and Y5 receptor ablation is the primary defect leading to hypophagia in our mouse models, the exact pathway leading to hypophagia is likely to be more complex than a simple lack of NPY signalling via Y1 and Y5 receptors. For instance, germline Y1Y5^−/−^ mice demonstrated decreased NPY mRNA expression in the hypothalamic arcuate nucleus. It was previously shown that NPY-ergic neurons in the arcuate nucleus inhibit the activity of neighboring POMC neurons, thereby dampening α-MSH-induced inhibition of food intake [37]. As such, reduced arcuate NPY expression in our Y1Y5 deficient mice could result in relative dis-inhibition of neighboring POMC neurons, which in itself could contribute to the observed hypophagia. It might be argued that the reduction in NPY expression in our Y1Y5^−/−^ mice is due to enhanced inhibitory action of the Y2 auto-receptor on these neurons, in keeping with the observation that deletion of Y1 receptors leads to up-regulation of Y2 receptor expression [38]. However, our Y receptor binding studies revealed no such evidence of Y2 receptor up-regulation in our Y1Y5 deficient mouse models, which on the other hand does not exclude increased activity. An additional potential contributor to the hypophagic phenotype of our Y1Y5 receptor deficient mice is concomitant obesity, which would be expected to induce hormonal changes known to reduce food intake, such as elevated circulating concentrations of leptin and the gut satiety hormone peptide YY [39]–[42]. However, obesity is not the only mechanism for hypophagia in our mice, because 18 week-old male Y1Y5^−/−^ mice on a chow diet exhibited clear hypophagia under both fasted and non-fasted conditions, despite the fact that they were not yet obese at this age.

Despite hypophagia, both germline and hypothalamus-specific Y1Y5 receptor knockout mice became obese. This was particularly apparent in the germline model, in which body weight was increased in mice on a normal chow diet from as young as 5 weeks of age onwards. In contrast, hypothalamus-specific knockout mice demonstrated increased body weight and/or adiposity only under conditions of high fat feeding, not on a normal chow diet. This early-onset obesity in germline knockouts may be due to compensatory responses to gene deletion, as has been observed in germline Y5 receptor knockout mice which exhibit exacerbated fasting-induced increases in hypothalamic expression of NPY and AgRP and exacerbated decreases in that of POMC and CART [18]. In keeping with the possibility of compensatory and obesogenic responses to gene deletion, male but not female Y1Y5^−/−^ mice exhibited significant reductions in energy expenditure, albeit hypothalamic arcuate nucleus (ARC) NPY mRNA levels were decreased and ARC POMC mRNA levels were unchanged in this model. One could hypothesise that up-regulation of other Y receptors may be compensating for the lack of Y1 and Y5 receptors in our knockout models, as was previously observed in Y1^−/−^ mice which exhibited increased Y2 receptor mRNA and protein levels compared to wildtype controls [38]. However, our binding studies revealed no change in total or Y2-specific binding in the arcuate nucleus of our Y1Y5 deficient mouse models, demonstrating that any adaptive changes contributing to obesity probably do not involve alterations in Y receptor expression. In the female Y1Y5^−/−^ mice, as well as in the hypothalamus-specific knockout model on a HFD, other changes besides reduced energy expenditure must have contributed to the exacerbated diet-induced obesity, as energy expenditure was either unchanged or significantly increased in these animals. Compensatory responses to dual Y1Y5 receptor ablation, whether germline or conditional, may originate from other central and peripheral systems that work with NPY to modulate energy balance. These include several other peptide and hormone signaling systems such as those of leptin, melanocortins, adiponectin and sex hormones [43]. Regardless of the underlying mechanisms, our observation of increased body weight and/or adiposity in Y1Y5 knockout mice despite decreased food intake and/or – in the case of older female Y1Y5^−/−^ mice and male Y1Y5^Hyp/Hyp^ mice on a HFD – increased energy expenditure, provide further support for the hypothesis that energy homeostatic systems are indeed biased towards the development of obesity [44].

Taken together, the findings from these studies demonstrate that dual deletion of Y1 and Y5 receptors, either globally or within the hypothalamus, results in marked and significant reductions in food intake, revealing a coordinated role of Y1 and Y5 in the regulation of appetite.
